# Problematic Internet Use and wellbeing during adolescence

**DOI:** 10.1192/j.eurpsy.2022.755

**Published:** 2022-09-01

**Authors:** J. Ortuño-Sierra, A. Pérez-Albéniz, A. Ciarreta, A. Díez Gómez, E. Fonseca Pedrero

**Affiliations:** 1 University of La Rioja, Educational Sciences, Logroño, Spain; 2 Univerity of La Rioja, Educational Sciences, Logroño, Spain

**Keywords:** mental health, Internet use, adolescence, wellbeing

## Abstract

**Introduction:**

PIU has not yet been recognized by diagnostic classification systems, but it has received increasing research and clinical attention. It is defined as a generalized and compulsive use of the Internet associated with a loss of control and negative consequences for the individual

**Objectives:**

The main goal was to analyze the relation between problematic Internet Use and wellbeing in adolescents

**Methods:**

The sample included a total of 1059 adolescents (47% were males). Age range was between 14 and 18 years old (M = 15,12; SD = 1,03). We used the Compulsive Internet Use Scale to assess Problematic Internet Use and the Strengths and Difficulties Questionnaire to screen for psychological difficulties and prosocial behaviour.

**Results:**

The results found in the ANOVA revealed that problematic internet use was statistically significant associated with psychological difficulties and prosocial capabilities (λ = 0.475, F(3,83,000) = 25.569, P ≤ 0.001, η² = 0.215).Adolescents with higher levels of Problematic Internet Use revealed more emotional and behavioural difficulties. In addition. those adolescents with higher levels of prosocial ablities were at a lower risk for Problematic Internet Use.

**Conclusions:**

Previous research have revealed that the use of Internet has almost doubled in the last decade among adolescents across different European countries. Results revealed statistically significant correlations between Problematic Internet Use and indicators of well-being such as emotional difficulties and behavioral problems, as well as prosocial behaviours. Prevention strategies should focus on detecting problematic internet use among adolescents, as it is a variable related with different psychological difficulties that are diminishing adolescents’ well-being.

**Disclosure:**

No significant relationships.

